# Lithium as a rescue therapy for regression and catatonia features in two SHANK3 patients with autism spectrum disorder: case reports

**DOI:** 10.1186/s12888-015-0490-1

**Published:** 2015-05-07

**Authors:** Sylvie Serret, Susanne Thümmler, Emmanuelle Dor, Stephanie Vesperini, Andreia Santos, Florence Askenazy

**Affiliations:** Autism Resources Center, University Child and Adolescent Psychiatry Department, Children’s Hospitals of Nice CHU-Lenval, CoBTek EA7276 University of Nice Sophia Antipolis, Nice, France

**Keywords:** Autism, *SHANK3* gene, Lithium, Regression, Catatonia

## Abstract

**Background:**

Phelan-Mc Dermid syndrome is a contiguous disorder resulting from 22q13.3 deletion implicating the *SHANK3* gene. The typical phenotype includes neonatal hypotonia, moderate to severe intellectual disability, absent or delayed speech, minor dysmorphic features and autism or autistic-like behaviour. Recently, point mutations or micro-deletions of the *SHANK3* gene have been identified, accompanied by a phenotype different from the initial clinically description in Phelan McDermid syndrome.

**Case presentation:**

Here we present two case studies with similar psychiatric and genetic diagnosis as well as similar clinical history and evolution. The two patients were diagnosed with autism spectrum disorders in childhood and presented regression with catatonia features and behavioural disorders after a stressful event during adolescence. Interestingly, both patients presented mutation/microdeletion of the *SHANK3* gene, inducing a premature stop codon in exon 21. Different pharmacological treatments (antipsychotics, benzodiazepines, mood stabilizer drugs, antidepressants, and methylphenidate) failed to improve clinical symptoms and lead to multiple adverse events. In contrast, lithium therapy reversed clinical regression, stabilized behavioural symptoms and allowed patients to recover their pre-catatonia level of functioning, without significant side effects.

**Conclusion:**

These cases support the hypothesis of a specific SHANK3 phenotype. This phenotype might be linked to catatonia-like deterioration for which lithium use could be an efficient treatment. Therefore, these cases provide an important contribution to the field of autism research, clinical genetics and possible pharmacological answers.

**Electronic supplementary material:**

The online version of this article (doi:10.1186/s12888-015-0490-1) contains supplementary material, which is available to authorized users.

## Background

Autism Spectrum Disorders (ASD) include a wide range of phenotypes represented by a continuum from slight to severe dysfunction of social interaction and communication as well as restrictive and stereotyped behaviour [1]. Among multiple etiologies of ASD, studies underlie a strong genetic component with mutations and deletions described in multiple genes [2]. The *SHANK3* gene on chromosome 22q13.3 appears to be of special interest as it is implicated in about 2% of ASD patients with intellectual deficiency [3]. SHANK3 is a post-synaptic scaffolding protein, involved in the regulation of the structural organization of dendritic spines, and a binding partner of neuroligins [4].

Deletions of chromosome region 22q13.3 including the *SHANK3* gene are responsible of the clinical manifestations of Phelan–McDermid syndrome (PMS) [5]. This syndrome is characterized by neonatal hypotonia, moderate to severe intellectual disability (ID), absent or delayed speech, minor dysmorphic features and autism or autistic-like behaviour. In addition, a clinically significant progressive loss of skills, atypical bipolar disorders, catatonia and behavioural disorders have been described [6-9]. The *SHANK3* gene is thought to play a major role for neurobehavioral phenotype, clinical evolution and neurological deterioration in PMS [7]. Interestingly, recent advances suggest that *SHANK3* mutations may lead to different phenotypes than PMS, which have not yet been clearly identified [3].

Here we describe two patients with ASD and a mutation/microdeletion of the *SHANK3* gene. Both patients presented regression, catatonia features and behavioural disorders. Importantly, clinical symptoms were reversed by lithium therapy, after failure and multiple side effects of other psychotropic medications (Additional files 1 and 2).

## Case presentations

### Case 1

Patient 1 is a 21-year-old man with ASD diagnosis based on the DSM-5 criteria as well as on the Autism Diagnostic Interview-Revised (ADI-R) [10] and the Autism Diagnostic Observation Schedule (ADOS) [11]. He is the second child of non-consanguineous parents and had no perinatal history. He was first evaluated at age 6 (see Table 1). At the verbal level, his language was limited to simple vocabulary of everyday life, with literal comprehension. He used words and short sentences to make a request and presented echolalia and stereotypic language. At the cognitive level, he has severe intellectual disability. At the motor level, he had no early psychomotor delay and liked to do sports and practiced biking for several hours a week. At age 13, as he moved from usual autism day-care centre to a non-specific autism unit, major clinical changes were observed.

**Table 1:** **Summary of patients’ early development and first evaluation (y: years, m: months)**

	**Patient 1**	**Patient 2**
**Diagnosis (DSM 5)**	**ASD**	**ASD**
**Early development**		
Perinatal history	No	No
Walking (y,m)	1,2	1,3
Cleanliness day and night (y)	3	3
Language (first words) (y,m)	3,5	2,3
**Age of first evaluation**	6	9
Autism intensity (CARS-T)	36	34.5
Intellectual level (Terman-Merill) (y,m)	2,9	2,9
Speech delay, echolalia, stereotypical language	yes	yes
Vineland I : Communication (y,m)	2,3	2,8
Daily living skills (y,m)	2,4	2,1
Socialization (y,m)	1,8	2,3

### From age 13 to 15: regression and behavioural disorders

In the following, patient 1 progressively reduced motor and verbal initiative, stopped his favourite activities, presented a language regression and lost autonomy skills. Additionally, behavioural disorders (impulsive acts, unpredictable gesture and opposition) alternated with apathy. At last, sleep disturbances and insomnia appeared.

### From age 15 to 19: catatonia symptoms and side effects of medications

At age 15, behavioural disorders required a hospitalization in an inpatient child and adolescent psychiatric unit. Neurological evaluation including brain Magnetic Resonance Imaging (MRI) and Electroencephalography (EEG) as well as biological analysis did not show any abnormalities. Genetic evaluation diagnosed a microdeletion of 4 bases in exon 21 of the *SHANK3* gene (c.3653_3656delCCCT), responsible of a premature stop codon (p.Ser1218CysfsX81).

Patient 1 received different psychotropic medications which were partially effective but accompanied by multiple side effects. (1) Antipsychotics were able to control aggressive behaviours and insomnia, but induced catatonia with positive evaluation by Bush Francis Catatonia Rating Scale (BFCRS) [12] and elevated muscle enzymes (creatine phosphokinase = 1286 UI/l, normal <175, and lactate dehydrogenase = 735 UI/l, normal: 200–480). (2) Benzodiazepines reduced catatonia symptoms but didn’t control impulsive actions, and induced verbal and motor excitement, confusion and insomnia. (3) Mood stabilizer drugs had no clinical efficacy and induced somatic problems such as cytolysis with valproic acid, and DRESS syndrome (Drug Reaction with Eosinophilia and Systemic Symptoms) with carbamazepine. (4) Antidepressants didn’t show any efficacy. (5) Among multiple drug associations only the co-therapy of aripiprazole and clonazepam showed partial efficacy improving regression, autonomy, language and motor skills for about 12 months, but without behaviour stabilization. (5) Methylphenidate was not effective and induced insomnia, violence and shouting. (6) Lithium was initially introduced in association with other psychotropic drugs (e.g. methylphenidate) and for a short period (<2 months), without clear clinical effects.

### From age 19 to 21: lithium therapy and reversion of symptoms

**Figure 1:**
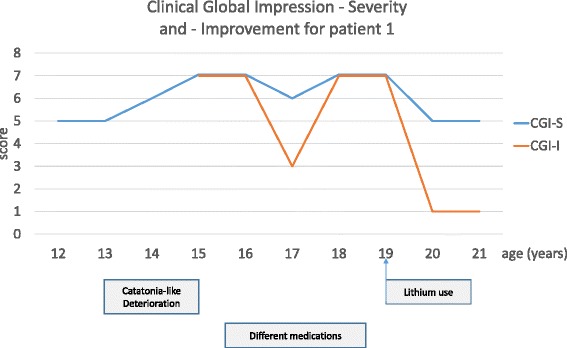
Clinical Global Improvement - Severity and - Improvement scale at different time points of the follow-up of patient 1. The Clinical Global Impression scale (CGI) has two components: the CGI-Severity (CGI-S), which is a seven-point scale rating severity of illness (1-normal to 7-extremely ill) and the CGI-Improvement (CGI-I), which assesses the change of patient’s illness since the initiation of treatment (1-very much improved to 7-very much worse) [18].

**Figure 2:**
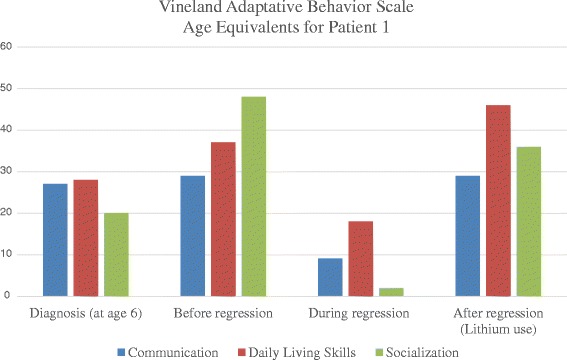
Vineland Adaptative Behavior Scale Age Equivalents for patient 1. The Vineland Adaptive Behavioral Scale (VABS) [19] was used to measure the level adaptive of functioning (Communication, Daily Living Skills and Socialization). VABS age equivalents are expressed in months (Y-axis) at different time points of the follow-up of patient 1 (diagnosis, before, during and after regression).

As there was no symptoms improvement, the diagnosis of atypical bipolar disorders was suspected. Therefore, it was decided to re-introduce lithium (1500 mg/day), only associated with melatonin (4 mg/day). In the following, symptoms progressively stabilized during the next 3 months. Agitation, hetero-aggressiveness and impulsivity disappeared. The patient regained autonomy in everyday life, and urinary and faecal incontinence stopped. In addition, the patient did no longer show opposition and participated again in different activities with other patients. Finally, he began riding his bike again. As the patient did no longer present sleep disturbances, melatonin could be stopped after 3 months of lithium therapy. With a follow-up of 2 years, lithium treatment permitted to reverse regression and to stabilize behavioural disorders and is well tolerated (1500 mg/day, blood lithium level =0.8 mEq/l, normal: 0.6-1). One year after the beginning of lithium therapy, the patient integrated a unit of life for adults. He recovered a level of functioning similar to the level he had before regression (see Figure 1 and Figure 2).

### Case 2

Patient 2 is a 17-year-old female adolescent with ASD diagnosis based on the DSM-5 criteria as well as on ADI-R and ADOS. She has first evaluation at age 9 (see Table 1). She is the only child of non-consanguineous parents without perinatal history. At the verbal level, she presented echolalia and stereotypic language with words and short sentences used in everyday life and in context. At the cognitive level, she has severe intellectual disability. At the motor level, she had no early psychomotor delay and she liked sports, especially rock climbing and gym. At age 12, major clinical changes were observed at the same time as she changed the day-care centre for a specific autism unit.

### From age 13 to 15: regression and behavioural disorders

After age 12, the patient presented regression and behavioural disorders. She lost night urinary autonomy, slowly reduced her initiative affecting motor and verbal skills, and developed impulsive and aggressive behaviours against other children. In the following, the patient modified her body posture getting vaulted and remained frequently motionless needing physical stimulation in order to be able to move. At last, she presented deterioration of skills and autonomy, urinary and faecal incontinence as well as sleep disturbances (insomnia). Brain MRI, EEG and biological analysis were normal. The genetic evaluation found a point mutation in exon 21 of the *SHANK3* gene (c.2425G > T) responsible of a premature stop codon (p.Glu809X).

### From age 15 to 16: catatonia features and side effects of medication

The clinical presentation suggested the diagnosis of catatonia-like symptoms without the full picture of catatonia (negative evaluation by BFCRS and normal blood analyses including muscle enzymes). In line with this hypothesis, we decided to introduce benzodiazepine treatment with clonazepam (0.9 mg/day). The patient rapidly improved and regained motor abilities, normal body posture, language, participation, and autonomy in a few months. Sleep disturbances and faecal incontinence stopped but night urinary incontinence persisted. These improvements continued for about 8 months. However, verbal and motor excitement progressively increased. Surprisingly, the patient progressively lost weight whereas she continued to ate normally (loss of 8 kg in 8 months) and without other clinical symptoms such as vomiting. Her initial body mass index (BMI) of 19.7 decreased to 16.4. We therefore decided to progressively withdrawal clonazepam. During the 4 following months, the patient regained weight (BMI =18.1), but then regressed again by losing motor and verbal skills. At last, she presented once more catatonia-like symptoms including behavioural disorders (hetero-aggressiveness) and insomnia. Clonazepam (0.9 mg/day) was re-introduced. Two days after, motor and verbal excitement, sudden impulsive actions, and total insomnia were observed requiring hospitalization in the inpatient psychiatric unit. Clonazepam was switched to lorazepam (3 mg/day), complicated by confusion, faecal incontinence, agitation and insomnia.

### From age 16 to 17: lithium therapy and reversion of symptoms

**Figure 3:**
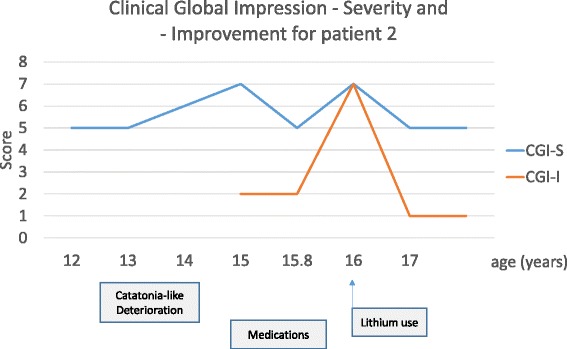
Clinical Global Improvement – Severity (CGI-S) and - Improvement scale (CGI-I) at different time points of the follow-up of patient 2.

**Figure 4:**
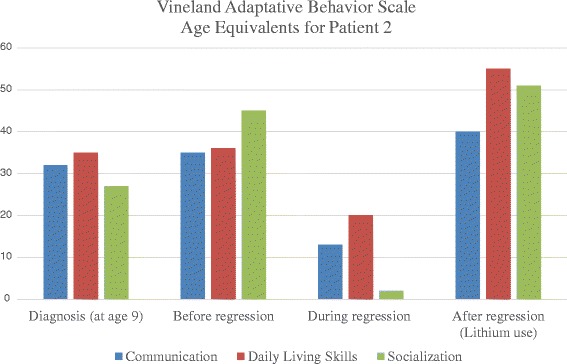
Vineland Adaptative Behavior Scale Age Equivalents for patient 2. VABS age equivalents are expressed in months (Y-axis) at different time points of the follow-up of patient 2 (diagnosis, before, during and after regression).

Based on the clinical similarities between patient 1 and 2, our clinical experience with patient 1 and the hypothesis of atypical bipolar disorder, we decided to introduce lithium, associated with very low dose clonazepam (0.2 mg/day). Patient 2 stabilized behavioural symptoms and progressively improved verbal and motor abilities, participation, sleep and autonomy. However, night urinary incontinence persisted. Clonazepam was discontinued after 4 months. After one year with lithium treatment (1000 mg/day, lithium blood level 0.7 mEq/l, normal 0.6-1), the patient recovered a similar level of functioning as before regression (see Figure 3 and Figure 4). Lithium reversed regression and stabilized behavioural disorders without side effects.

## Conclusion

The two patients described in this article had a similar psychiatric (ASD) and genetic diagnosis (mutation/microdeletion of the *SHANK3* gene inducing a premature stop codon in exon 21) as well as similar clinical, history and evolution (regression with catatonia features after a stressful event in adolescence). In contrast, multiple psychotropic medications were partially or not effective and accompanied by important side effects. Finally, lithium therapy permitted reversibility of regression and catatonia, and the stabilization of behavioural disorders without significant side effects.

Unlike most clinical descriptions of patients presenting *SHANK3* deletions and Phelan–McDermid syndrome, our patients present a somewhat different phenotype which might be linked to the size and location of the molecular anomalies [3,13]. There was no history of hypotonia and the motor level permitted the regular practice of sports [5]. The progressive loss of skills (verbal, motor and autonomy) associated with catatonia features, behavioural disorders and sleep disturbances is suggestive of “catatonia-like deterioration” described in ASD patients after a stressful event [14].

Nevertheless, we describe for the first time two cases for which reversibility of those symptoms was found after lithium treatment. Lithium is a mood stabilizer drug with a long history in psychiatry. It is mainly used in adult patients to treat manic episodes in bipolar disorder and for treatment-resistant depression [15]. Our finding of clinical efficacy of lithium is in line with the recent idea that *SHANK3* patients with ASD may present an atypical form of bipolar disorders [8,9]. Importantly, lithium therapy was proposed after several therapeutic failures leading to severe side effects. Indeed, studies suggest that *SHANK3* ASD patients might be more vulnerable to psychotropic drugs than other ASD patients [7]. In addition, lithium has been identified having an action on synaptic modulation with a possible neuroprotective role in neurodegenerative diseases [16]. Lithium might be a rescue therapy for some patients presenting shankopathies by partially reversing impaired synaptic function and neural circuit defects [17]. Further clinical and biological studies are required to explore this hypothesis.

The current report underlines the importance of building bridges between genetics and clinical follow-up, not only for further understanding of genetic psychiatric disorders but also for treatment decisions. While this report presents limitations (two cases and after only one year), it opens new avenues to the study of shankopathies for which phenotype descriptions and treatment recommendations are still sparse.

## Consent

Patients’ legal guardians have given their written consent for this case reports being published.

## Additional files

Additional file 1:
**CARE Checklist (2013).**
Additional file 2:
**Timeline.**

## Abbreviations

ADI-R: Autism diagnostic interview-revised
ADOS: Autism diagnostic observation schedule
ASD: Autism spectrum disorders
BFCRS: Bush-Francis catatonia rating scale
BMI: Body mass index
CARS: Childhood autism rating scale
CGI-S: Clinical global impression - severity
CGI-I: Clinical global impression - improvement
DRESS: Drug reaction with eosinophilia and systemic symptoms
EEG: Electroencephalography
ID: Intellectual disability
MRI: Magnetic resonance imaging
PMS: Phelan–McDermid syndrome
VABS: Vineland adaptative behavioural scale
