# Supplementary material for: Lithium as a rescue therapy for regression and catatonia features in two SHANK3 patients with autism spectrum disorder: case reports
Source: BMC Psychiatry. 2015 May 7;15:107. doi: 10.1186/s12888-015-0490-1 (PMC4428105; doi:10.1186/s12888-015-0490-1)
Supplement: Additional file 1: — CARE Checklist (2013). [file 12888_2015_490_MOESM1_ESM.pdf]

CARE CHECKLIST of information to include when writing a case report

Section	Item	Checklist item description	Reported on page
**Title**	1	The words "case report" and the area of focus should appear in the title (such as diabetes, a therapeutic approach, an outcome)	P 1
**Key Words**	2	2 to 5 key words that identify areas covered in this case report	P 3
**Abstract**	3a	Introduction—What is unique about this case? What does it add to the medical literature? Why is this important?	P4
	3b	The patient's main concerns and important clinical findings	P 3
	3c	The main diagnoses, therapeutics interventions, and outcomes	P 2,3
	3d	Conclusion—What are the "take-away" lessons from this case?	P 3
**Introduction**	4	One or two paragraphs summarizing why this case is unique with reference to the relevant medical literature	P 4
**Patient Information**	5a	De-identified demographic and other patient specific information	P 4,6
	5b	Main concerns and symptoms of the patient	Fig. 1, 2, 3 and 4
	5c	Medical, family, and psychosocial history including relevant genetic information (this should also appear in the timeline)	P 4,5,6,7
	5d	Relevant past interventions and their outcomes	P 5,7
**Clinical Findings**	6	Describe the relevant physical examination (PE) and other significant clinical findings	P 4,6
**Timeline**	7	Relevant data from the patient's history organized as a timeline	Fig. 2,4
**Diagnostic Assessment**	8a	Diagnostic methods (PE, laboratory testing, imaging, surveys)	P 5,7
	8b	Diagnostic challenges (access, financial, cultural)	P 3
	8c	Diagnostic reasoning including other diagnoses considered	P 6,8
	8d	Prognostic characteristics when applicable (staging)	P 6
**Therapeutic Intervention**	9a	Types of intervention (pharmacologic, surgical, preventive)	P 6,8
	9b	Administration of intervention (dosage, strength, duration)	P 6,8
	9c	Any changes in the interventions (with rationale)	P 6,8
**Follow-up and Outcomes**	10a	Clinician and patient-assessed outcomes (when appropriate)	Fig. 2,4
	10b	Important follow-up diagnostic and other test results	Fig 1, 2, 3 and 4
	10c	Intervention adherence and tolerability (how was this assessed)	Fig. 1 and 3
	10d	Adverse and unanticipated events	P 6,8
**Discussion**	11a	Strengths and limitations in your approach to this case	p 9
	11b	Discussion of the relevant medical literature	p 8-9
	11c	The rationale for your conclusions (a causality assessment)	Fig. 2 and 4
	11d	The primary "take-away" lessons from this case report	p 9
**Patient Perspective**	12	When appropriate the patient should share their perspective on the treatments they received	P 9

**Informed Consent**	13	Did the patient give informed consent? Please provide if requested	Yes
