# Supplementary figures and images for: Lithium as a rescue therapy for regression and catatonia features in two SHANK3 patients with autism spectrum disorder: case reports
Source: BMC Psychiatry. 2015 May 7;15:107. doi: 10.1186/s12888-015-0490-1 (PMC4428105; doi:10.1186/s12888-015-0490-1)

## Patient 1

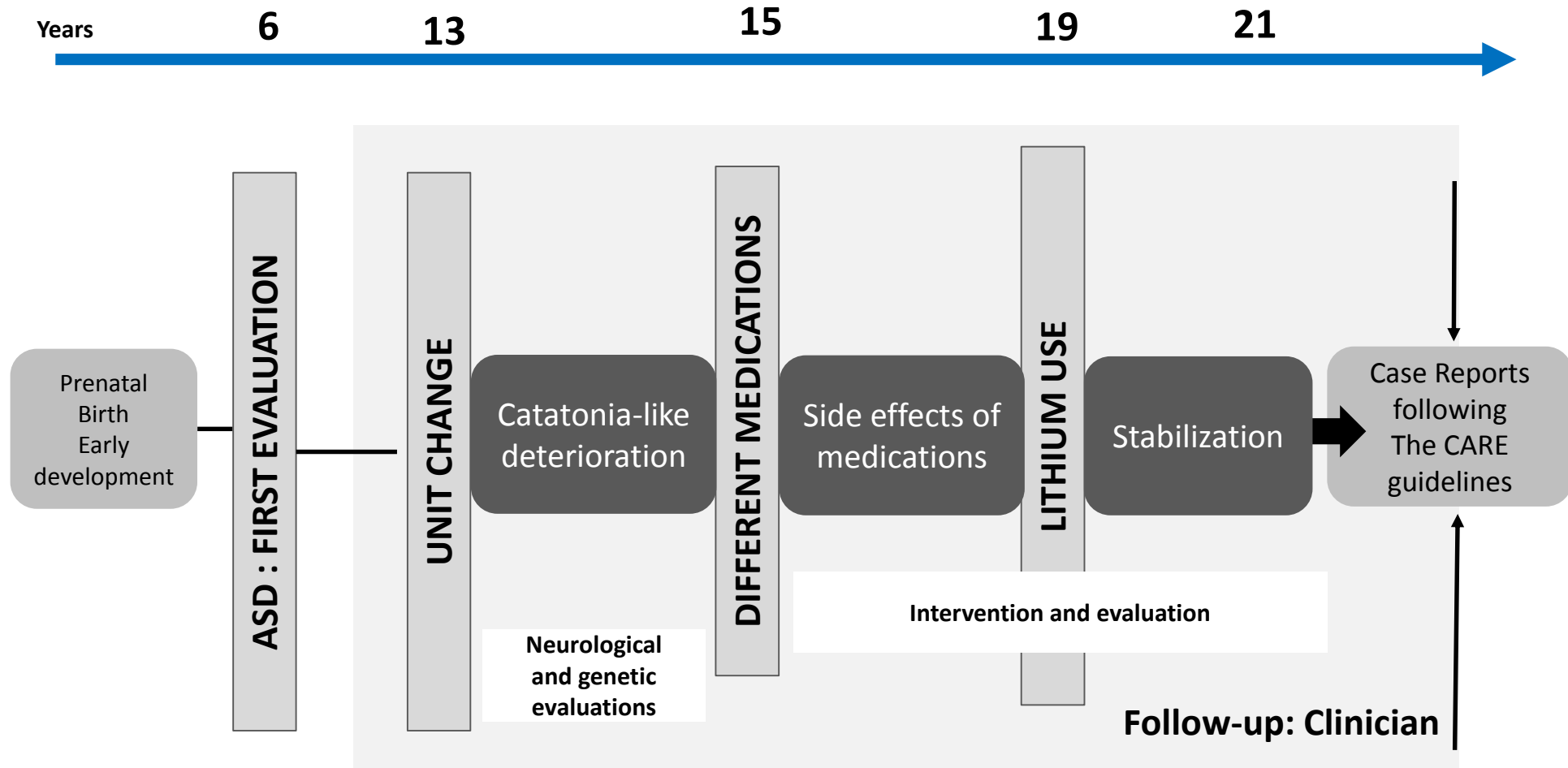

## Patient 2

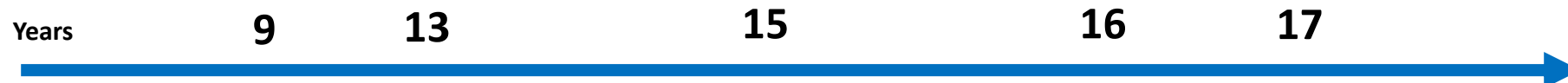

Supplement: Additional file 2: — Timeline. [file 12888_2015_490_MOESM2_ESM.pdf]
